# Heating ability of elongated magnetic nanoparticles

**DOI:** 10.3762/bjnano.12.104

**Published:** 2021-12-28

**Authors:** Elizaveta M Gubanova, Nikolai A Usov, Vladimir A Oleinikov

**Affiliations:** 1 National Research Nuclear University “MEPhI”, 115409, Moscow, Russia; 2 Pushkov Institute of Terrestrial Magnetism, Ionosphere and Radio Wave Propagation, Russian Academy of Sciences, IZMIRAN, 108480, Troitsk, Moscow, Russia

**Keywords:** elongated magnetic nanoparticles, magnetic hyperthermia, numerical simulation, specific absorption rate

## Abstract

Low-frequency hysteresis loops and specific absorption rate (SAR) of various assemblies of elongated spheroidal magnetite nanoparticles have been calculated for a range of particle semiaxis ratios *a*/*b* = 1.0–3.0. The SAR of a dilute randomly oriented assembly of magnetite nanoparticles in an alternating magnetic field of moderate frequency, *f* = 300 kHz, and amplitude *H*_0_ = 100–200 Oe is shown to decrease significantly with an increase in the aspect ratio of nanoparticles. In addition, there is a narrowing and shift of the intervals of optimal particle diameters towards smaller particle sizes. However, the orientation of a dilute assembly of elongated nanoparticles in a magnetic field leads to an almost twofold increase in SAR at the same frequency and amplitude of the alternating magnetic field, the range of optimal particle diameters remaining unchanged. The effect of the magneto-dipole interaction on the SAR of a dilute assembly of oriented clusters of elongated magnetite nanoparticles has also been investigated depending on the volume fraction of nanoparticles in a cluster. It has been found that the SAR of the assembly of oriented clusters decreases by approximately an order of magnitude with an increase in the volume fraction of nanoparticles in a cluster in the range of 0.04–0.2.

## Introduction

Magnetic nanoparticle assemblies have great potential for the use in biomedicine, in particular, in magnetic hyperthermia [1–4], a new promising approach for cancer treatment. In this method, magnetic nanoparticles introduced into a tumor and excited by an alternating (ac) low-frequency magnetic field are able to warm up malignant tissues locally. In most cases this stops the tumor growth and results in its decay. However, it are magnetic nanoparticles with low toxicity and a high specific absorption rate (SAR) of the energy of the ac magnetic field that are needed in magnetic hyperthermia. The use of optimized assemblies of magnetic nanoparticles can significantly reduce the concentration of particles introduced into the tumor to obtain a positive therapeutic effect. In recent years, a significant amount of works [5–17] has been devoted to the creation of various assemblies of magnetic nanoparticles suitable for magnetic hyperthermia. Basically, iron oxide nanoparticles were studied [5–10] because of their low toxicity and high saturation magnetization, although nanoparticles of other chemical compositions, such as metallic iron nanoparticles [11–13], and various ferrites [14–17] were also examined.

Theoretical studies [18–26] show that to achieve high SAR values several important factors have to be taken into account, such as the geometric dimensions of particles, particle saturation magnetization, magnitude of the magnetic anisotropy constant, and concentration and spatial distribution of nanoparticles in a tumor. Nevertheless, which type of magnetic nanoparticles might be most effective in magnetic hyperthermia has been the subject of debates so far [1–4,24,27]. For example, the use of magnetic nanoparticles with increased value of magnetic anisotropy constant was assumed [28–29] to be promising in magnetic hyperthermia. However, numerical simulations [30] showed that for spherical nanoparticles an increase of the uniaxial anisotropy constant leads to a decrease in SAR and a shift of the optimal particle diameters to smaller dimensions.

Another idea is to use elongated magnetic nanoparticles [9,31–36], which in a number of experiments [31,33] showed a rather high SAR. It is well known that elongated magnetic nanoparticles can be easily oriented in external magnetic fields. Furthermore, oriented assemblies of nanoparticles showed increased SAR [37–38] when the ac magnetic field was applied parallel to the assembly orientation axis. It is worth mentioning that, in nature, some strains of magnetotactic bacteria can synthesize elongated magnetic nanoparticles [39–41]. Biogenic magnetic nanoparticles have a perfect crystal structure and narrow size distribution, which makes them potential agents for magnetic hyperthermia [27,42].

In this work, by using the stochastic Landau–Lifshitz equation [43–46], we calculate low-frequency hysteresis loops and SAR of assemblies of elongated spheroidal magnetite nanoparticles with aspect ratio *a*/*b* > 1. Although in some experimental works [31,33] it has been argued that an increase in the particle aspect ratio contributes to an increase in SAR, our calculations show that the change in the magnetic properties of an assembly as a function of the particle aspect ratio is more complex. Namely, it is found that the SAR of a dilute randomly oriented assembly of magnetite nanoparticles in an ac magnetic field of moderate frequency, *f* = 300 kHz, and amplitude, *H*_0_ = 100–200 Oe, significantly decreases with an increase in the particle aspect ratio. In addition, there is a narrowing and shift of the intervals of optimal particle diameters towards smaller sizes. In contrast, for oriented assembly of elongated nanoparticles a significant, almost twofold increase in SAR is found at the same frequency and ac magnetic field amplitude, the range of optimal particle diameters being unchanged.

We also studied the effect of magneto-dipole (MD) interactions on the SAR of a dilute assembly of oriented clusters of elongated magnetite nanoparticles. In agreement with previous studies [23,25], the SAR of a dilute assembly of oriented clusters is shown to decrease by approximately 7–8 times with an increase in the particle volume fraction in a cluster in the range η = 0.04–0.2.

## Numerical Simulation

In this work the magnetic properties of various assemblies of elongated spheroidal magnetite nanoparticles with different aspect ratios *a*/*b* > 1 are studied by means of numerical simulation. It is assumed for simplicity that single-domain magnetite nanoparticles are monocrystalline, so that the cubic-type magneto-crystalline anisotropy energy of the assembly is given by [25]:


[1]

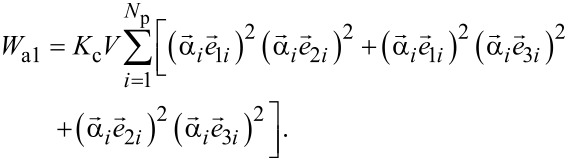



**Figure 1:**
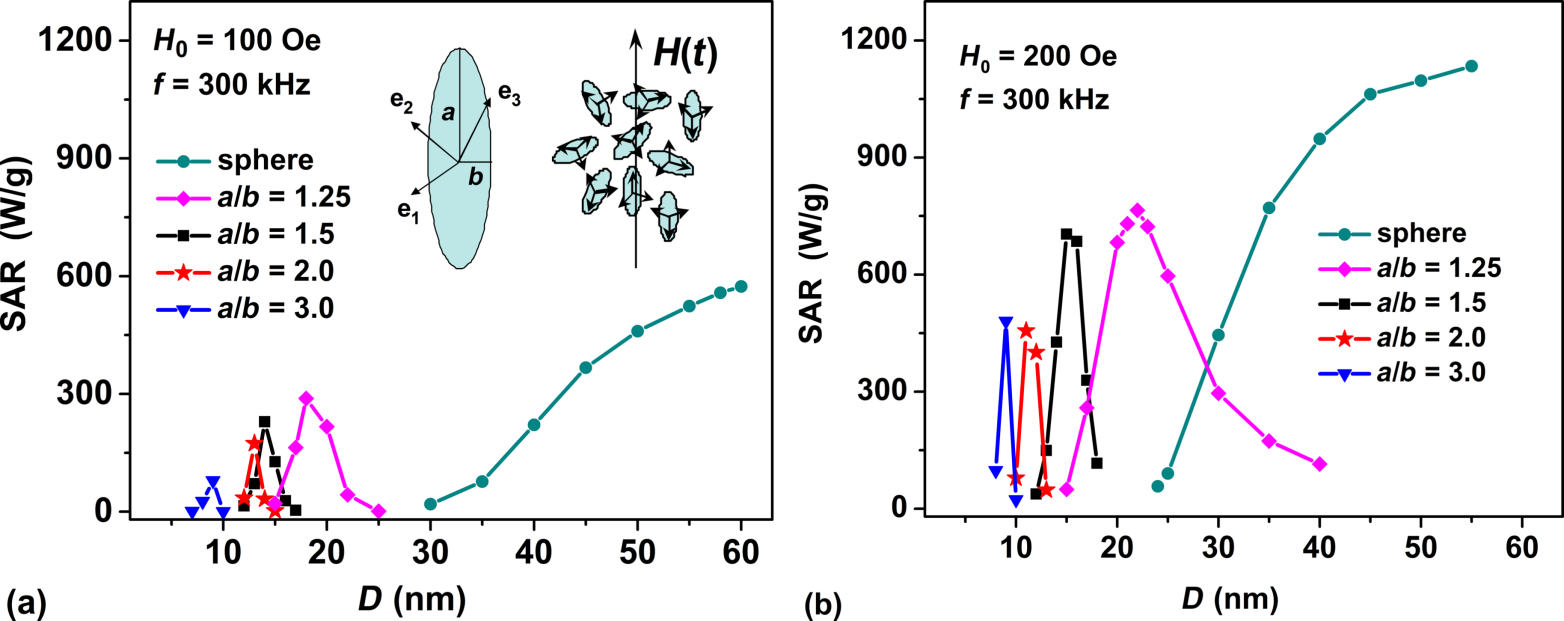
Dependence of SAR of a dilute randomly oriented assembly of magnetite nanoparticles with different aspect ratio *a*/*b* on the transverse particle diameter *D* = 2*b* at a frequency *f* = 300 kHz and at different amplitudes of the ac magnetic field: (а) *H*_0_ = 100 Oe; (b) *H*_0_ = 200 Oe.

Here, *K*_c_ = −10^5^ erg/cm^3^ is the cubic magnetic anisotropy constant [47], *V* = π*ab*^2^/6 is the volume of a spheroidal particle, 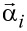 is the unit magnetization vector and (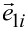, 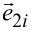, 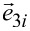) is the set of orthogonal unit vectors that determine the spatial orientations of the cubic easy anisotropy axes of the *i*-th nanoparticle of the assembly, *N*_p_ being the number of nanoparticles. It is also assumed that the axes of symmetry of spheroidal nanoparticles are arbitrarily directed with respect to the particle easy anisotropy axes (see below the inset in Figure 1a). Then, the shape anisotropy energy of the assembly of spheroidal nanoparticles can be written as follows:


[2]

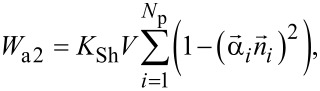



where *K*_Sh_ is the shape anisotropy constant and 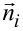 is the unit vector along the direction of elongation of the *i*-th nanoparticle. The shape anisotropy constant is given by [48]:


[3] $$\begin{array}{c} K_{\text{Sh}}=M_{\text{s}}^{2}\left(\pi -3N_{\text{a}}/4\right);\\ N_{\text{a}}=2\pi \frac{1-\varepsilon^{2}}{\varepsilon^{3}}\left(\ln\frac{1+\varepsilon }{1-\varepsilon }-2\varepsilon \right);\\ \varepsilon =\sqrt{1-{\left(b/a\right)}^{2}}. \end{array}$$

Here *M*_s_ = 450 emu/cm^3^ is the saturation magnetization of a magnetite nanoparticle [47] and *N*_a_ is the demagnetizing factor along the long nanoparticle axis.

In this work, we also consider the effect of MD interaction on the SAR of a dilute assembly of oriented clusters consisting of spheroidal magnetite nanoparticles. To calculate the energy of MD interaction between single-domain spheroidal particles of an oriented cluster we used the general formula [49]:


[4]

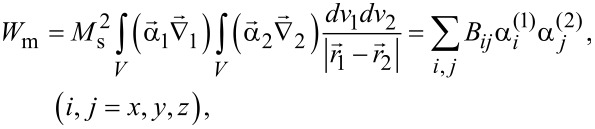



where *B_ij_* are the elements of the MD interaction matrix. For a pair of oriented spheroidal nanoparticles with an aspect ratio *a*/*b* > 1 the expressions for nonzero elements of the MD interaction matrix *B_xx_*, *B_уу_*, *B_zz_*, and *B_xz_* were obtained [50] numerically depending on the distance between the nanoparticle centers.

The calculation of low-frequency hysteresis loops and SAR of assemblies of elongated magnetite nanoparticles is carried out by solving the stochastic Landau–Lifshitz equation, according to the same scheme that was used in our previous works [23,25]. Using this approach one can take into account both the thermal fluctuations of particle magnetic moments at a finite temperature *T* and the influence of MD interaction in dense clusters of magnetic nanoparticles. The thermal fields 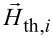, *i* = 1, 2,…, *N*_p_, acting on various nanoparticles of the cluster are statistically independent. Their components have the following statistical properties [43]:


[5] $$\begin{array}{c} \langle H_{\text{th}.i}^{\left(\alpha \right)}\left(t\right)\rangle =0;\\ \langle H_{\text{th},i}^{\left(\alpha \right)}\left(t\right)H_{\text{th},i}^{\left(\beta \right)}\left(t_{1}\right)\rangle =\frac{2k_{\text{B}}T\kappa }{\gamma M_{\text{s}}V}\delta_{\alpha \beta }\delta \left(t-t_{1}\right),\\ \alpha ,\beta =\left(x,y,z\right). \end{array}$$

Here *k*_B_ is the Boltzmann constant, γ is the gyromagnetic ratio, κ is phenomenological damping constant, δ_αβ_ is the Kronecker delta, and δ(*t*) is the delta function. The calculations of the low-frequency hysteresis loops are carried out at room temperature, *T* = 300 K.

## Results and Discussion

### SAR of dilute randomly oriented assemblies

Let us first consider the case of randomly oriented assembly of non-interacting elongated magnetite nanoparticles, assuming that the directions of the symmetry axes of spheroidal nanoparticles are nearly uniformly distributed in space. Figure 1a and Figure 1b show the dependence of the SAR on the transverse particle diameter *D* = 2*b* for assemblies with aspect ratios in the range *a*/*b* = 1.0–3.0 at a typical frequency *f* = 300 kHz, and at different amplitudes of the ac magnetic field *H*_0_ = 100 and 200 Oe, respectively.

As Figure 1 shows, the SAR of these assemblies substantially depends on both the transverse diameter *D* and the particle aspect ratio. It is easy to see that for a given aspect ratio, there is an optimal range of transverse particle diameters, in which the SAR of the assembly reaches maximum. Moreover, with an increase in the aspect ratio the following trends are observed: (a) a decrease in the maximum SAR of the assembly, (b) a shift of the window of optimal particle diameters to a range of smaller sizes, and (c) a significant decrease in the width of these windows for particles with large aspect ratios. Indeed, as Figure 1 shows, for spherical nanoparticles, *a*/*b* = 1.0, at the amplitude *H*_0_ = 100 Oe the maximal SAR values are sufficiently high, of the order of 450–600 W/g. Furthermore, the SAR of spherical nanoparticles increases up to 950–1100 W/g at *H*_0_ = 200 Oe. In addition, the range of optimal diameters of spherical particles turns out to be as wide as possible, *D* = 45–60 nm. At the same time, for particles with the aspect ratio *a*/*b* = 3.0 the domain of optimal particle diameters becomes extremely narrow, *D* = 8–10 nm, and the SAR maximum significantly decreases in comparison with that for the assembly of spherical magnetite nanoparticles.

It is well known that the SAR of an assembly of magnetic nanoparticles is proportional to the area of the low-frequency hysteresis loop of the assembly. It can be calculated [18,20] by the formula SAR = 10^−7^*M*_s_*fA*/ρ (W/g), where *A* is the hysteresis loop area in the variables (*M*/*M*_s_, *H*), and ρ is the density of magnetite nanoparticles, which is assumed to be ρ = 5 g/cm^3^.

**Figure 2:**
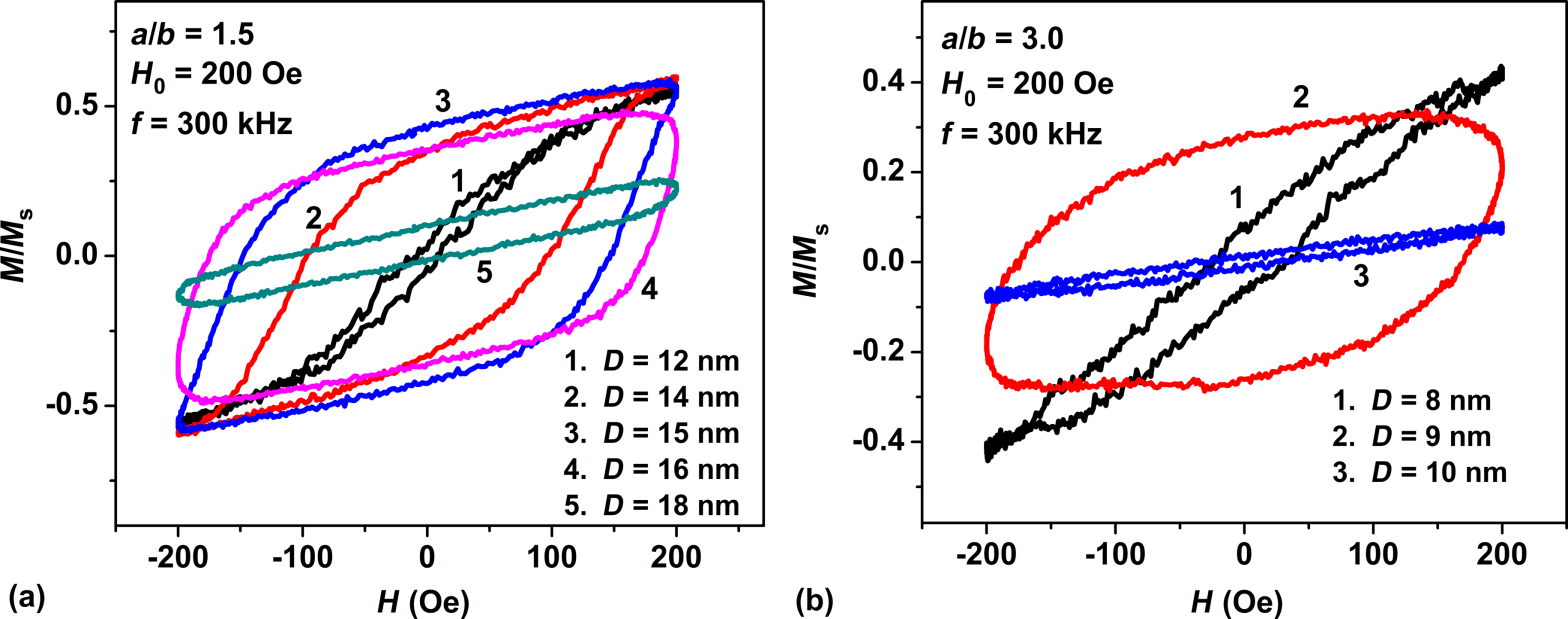
Evolution of the low-frequency hysteresis loops of dilute randomly oriented assemblies of magnetite nanoparticles as a function of the transverse particle diameter *D* for different aspect ratios of particles: (a) *a*/*b* = 1.5; (b) *a*/*b* = 3.0.

Figure 2 shows the evolution of low-frequency hysteresis loops of non-interacting randomly oriented assemblies of magnetite nanoparticles with aspect ratios *a*/*b* = 1.5 and 3.0 depending on the transverse particle diameter *D* = 2*b*. One can see that for particles with a large aspect ratio, *a*/*b* = 3.0, the area of the low-frequency hysteresis loop changes extremely rapidly with a change of the transverse diameter in the range of 8–10 nm.

The explanation of the observed behavior of the SAR of non-interacting assembly of elongated nanoparticles is straightforward. For magnetite nanoparticles with a high saturation magnetization, the shape anisotropy energy of the particle rapidly increases with increasing aspect ratio. For example, for magnetite a nanoparticle with aspect ratio *a*/*b* = 1.25 the shape anisotropy constant is *K*_Sh_ = 1.09 × 10^5^ erg/cm^3^, but this value increases to *K*_Sh_ = 4.29 × 10^5^ erg/cm^3^ for a particle with aspect ratio *a*/*b* = 3.0. Consequently, the uniaxial shape anisotropy energy dominates in the total anisotropy energy of magnetite nanoparticles with a sufficiently large aspect ratio, *a*/*b* > 1.5. The effective particle anisotropy constant, *K*_ef_ ≈ *K*_Sh_, determines the characteristic value of the reduced energy barrier between potential wells. The latter is equal in order of magnitude to σ = *K*_ef_*V*/*k*_B_*T*, where *k*_B_ is the Boltzmann constant and *T* is the absolute temperature. The dynamics of the unit magnetization vector of a superparamagnetic nanoparticle substantially depends on the value of the reduced energy barrier [18]. At a large value of the reduced energy barrier, σ ≫ 1, the probability of a nanoparticle magnetization reversal in an ac magnetic field of moderate amplitude, *H*_0_ ≪ *H*_k_, where *H*_k_ = 2*K*_ef_/*M*_s_ is the particle anisotropy field, is very small. At the same time, at small values of this parameter, σ ≪ 1, the dynamics of the unit magnetization vector is determined by thermal fluctuations, and the effect of the ac magnetic field is insignificant. Therefore, at fixed amplitude of the ac magnetic field, with increase in the effective anisotropy constant *K*_ef_ the magnetization reversal process is only possible for nanoparticles with a reduced volume. This fact explains the shift of the domain of optimal particle diameters to smaller sizes with an increase in the aspect ratio *a*/*b*. Simultaneously, a narrowing of the window of optimal diameters occurs, since with an increased value of *K*_ef_, the height of the reduced energy barrier changes rapidly with a relatively small change in the particle volume.

Note that earlier [30] a similar behavior of SAR was revealed for dilute randomly oriented assemblies of spherical iron oxide nanoparticles with saturation magnetization *M*_s_ = 350 emu/cm^3^ depending on the value of the effective uniaxial magnetic anisotropy constant in the range *K*_u_ = 1 × 10^4^–5 × 10^5^ erg/cm^3^. This fact shows once again that the dependence of the SAR of a dilute assembly of magnetite nanoparticles on the aspect ratio is associated with the dominant contribution of the shape anisotropy energy in the total anisotropy energy of particles with a sufficiently large aspect ratio, *a*/*b* > 1.5.

### SAR of dilute oriented assemblies

It follows from the results obtained in the previous section that the use of assemblies of magnetite nanoparticles with large aspect ratios in magnetic hyperthermia, generally speaking, is unfavorable, since an increase in the particle aspect ratio leads not only to a decrease in the SAR of the assembly, but also to a corresponding decrease in the width of the window of optimal particle diameters. However, it was shown experimentally [37–38] that the SAR of an assembly can be significantly increased if the assembly is oriented in a strong magnetic field and an ac magnetic field is applied along the assembly orientation axis. Recent study shows [17] that, under certain conditions, the orientation of the assembly is possible both in vitro and in vivo.

Let us assume that the axes of symmetry of spheroidal nanoparticles are oriented in a viscous liquid under the action of a strong permanent external magnetic field. Evidently, the orientation of the long particle axes can be fixed by increasing significantly the viscosity of the medium where the nanoparticles are distributed. However, the orientation of the cubic easy anisotropy axes of magnetite nanoparticles can hardly be changed. In the calculations performed it is supposed to be random as before.

**Figure 3:**
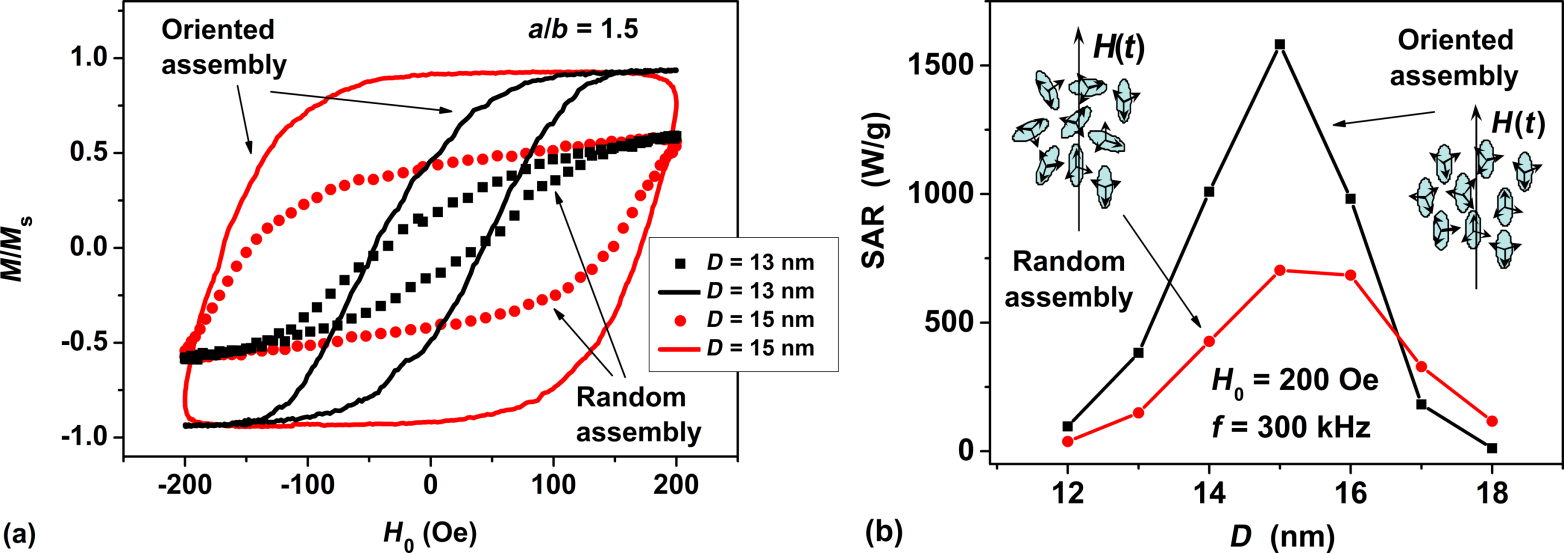
(a) Comparison of low-frequency hysteresis loops of dilute oriented (solid lines) and non-oriented (dots) assemblies of nanoparticles with aspect ratio *a*/*b* = 1.5 and diameters *D* = 13 and 15 nm. (b) SAR of oriented and non-oriented dilute assemblies of magnetite nanoparticles with aspect ratio *a*/*b* = 1.5 depending on the transverse particle diameter.

In Figure 3a solid curves show the low-frequency hysteresis loops calculated numerically for oriented assemblies of non-interacting nanoparticles with aspect ratio *a*/*b* = 1.5. The transverse particle diameters are given by *D* = 13 and 15 nm, respectively. The ac magnetic field with frequency *f* = 300 kHz and amplitude *H*_0_ = 200 Oe is applied along the assembly orientation axis, so that the angle between the ac magnetic field direction and the assembly orientation axis is zero, θ = 0. For comparison, in the same figure filled squares and filled circles show hysteresis loops of dilute randomly oriented assemblies of nanoparticles of the same diameters.

Figure 3a demonstrates that for assemblies of both types the shape of the hysteresis loops depends significantly on the transverse particle diameter *D* = 2*b*. It is interesting to note that the areas of the hysteresis loops reach a maximum at the same value of the transverse particle diameter, *D* = 15 nm. However, the hysteresis loop area of the oriented assembly is significantly larger than that of the non-oriented one. Accordingly, as Figure 3b shows, the SAR of the oriented assembly at the maximum is approximately two times higher than that of the non-oriented assembly. Nevertheless, for both types of assemblies the window of optimal particle diameters remains approximately the same.

### Influence of MD interaction

It was shown theoretically and experimentally [22–26,51–53] that the SAR of an assembly of magnetic nanoparticles is significantly reduced under the influence of strong MD interaction of the particles in a sufficiently dense assembly. It is known [54] that dense clusters of magnetic nanoparticles usually spontaneously form in biological cells and intercellular space upon the introduction of nanoparticles into tissue. In this section the SAR of a dilute assembly of oriented clusters of spheroidal magnetite nanoparticles is investigated as a function of volume fraction of nanoparticles in the clusters. The particle volume fraction can be defined [23] as η = *N*_p_*V*/*V*_cl_, where *N*_p_ is the average number of particles in a cluster and *V*_cl_ is the volume of spherical cluster. In a random cluster, nanoparticle centers are randomly distributed in the cluster volume. To construct an assembly of oriented clusters of spheroidal nanoparticles with randomly located nanoparticle centers, a modification of the algorithm described in [23] was used. Evidently, to place randomly in space two spheroidal nanoparticles with semiaxes *a* > *b*, the long axes of which are parallel to the *Z*-axis of the Cartesian coordinates, it is sufficient to require that the distances *L_x_*, *L_y_*, and *L_z_* between the centers of particles along the *X*-, *Y*-, and *Z*-axes satisfy the conditions *L_x_*, *L_y_* ≥ 2*b* and *L_z_* ≥ 2*a*.

In the present work, the low-frequency hysteresis loops are calculated for dilute assemblies of oriented quasispherical clusters containing *N*_p_ = 60–90 spheroidal magnetite nanoparticles with different aspect ratios. To obtain statistically reliable data, the low-frequency hysteresis loops are averaged over a sufficiently large number *N*_exp_ = 20–30 of clusters with independent implementation.

**Figure 4:**
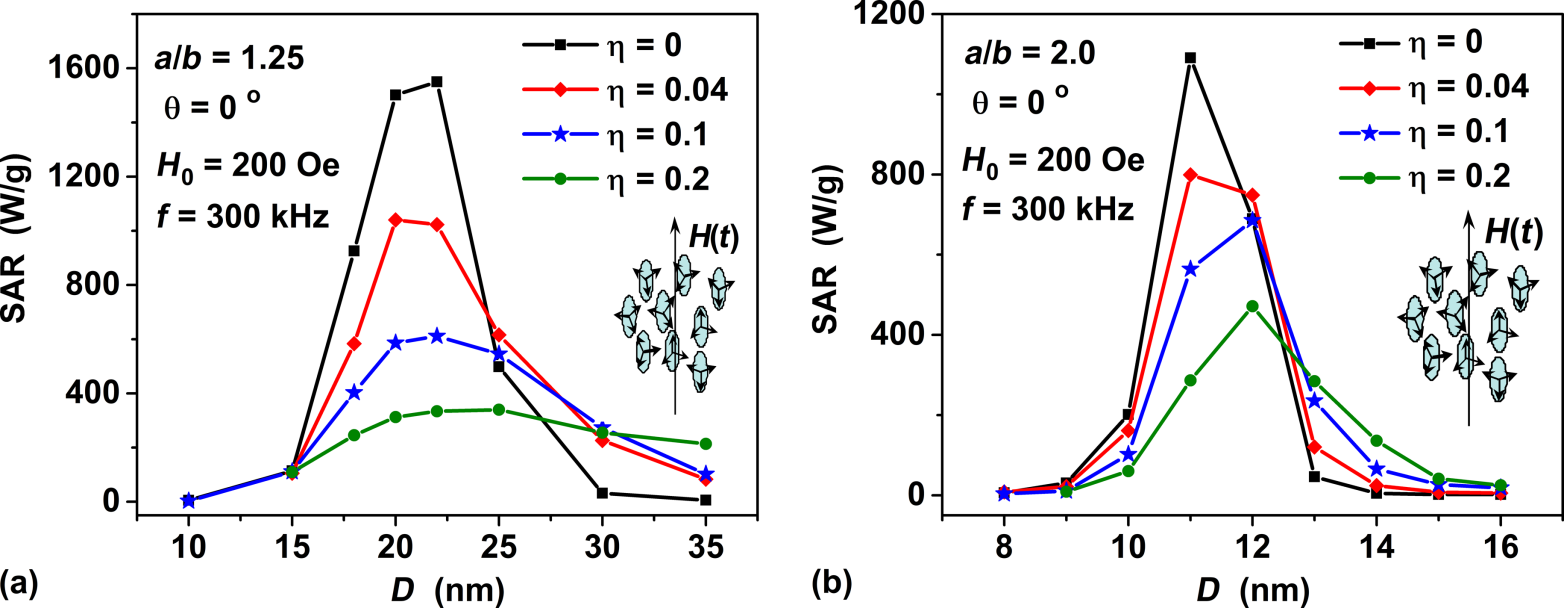
Dependence of SAR of a dilute oriented assembly of clusters of spheroidal magnetite particles with different aspect ratios (a) *a*/*b* = 1.25, and (b) *a*/*b* = 2.0 on the transverse particle diameter *D* = 2*b* at different volume fractions of nanoparticles η; the ac magnetic field is directed along the particle orientation axis, θ = 0.

Figure 4a shows the dependence of the SAR of a dilute assembly of oriented clusters of spheroidal nanoparticles with aspect ratio *a*/*b* = 1.25 on the transverse particle diameter for different volume fractions of nanoparticles in a cluster, η = 0.04–0.2, in comparison with an oriented assembly of non-interacting nanoparticles, η = 0. According to Figure 4a, for given ac magnetic field parameters the SAR of the oriented assembly of non-interacting nanoparticles can reach a maximum of about 1500–1600 W/g. However, with an increase in the volume fraction of particles in the cluster, the SAR values at the maximum rapidly decrease, so that at η = 0.2 they fall to values of the order of 250–330 W/g. The dependence of the SAR of a dilute oriented assembly of clusters of spheroidal magnetite particles on the volume fractions of nanoparticles in the cluster is an evident consequence of MD interaction among the particles. The intensity of the MD interaction increases as a function of the volume fraction η, because the average distance between the particle centers is roughly proportional to 1/η^1/3^.

It is interesting to note that with a change in the cluster volume fraction, the window of optimal particle diameters remains approximately constant, *D* = 17–24 nm. As Figure 4b shows, a similar behavior of the SAR value is observed for an assembly of oriented clusters of nanoparticles with aspect ratio of *a*/*b* = 2.0, with the difference that the window of optimal particle diameters in this case narrows to the interval *D* = 11–13 nm.

**Figure 5:**
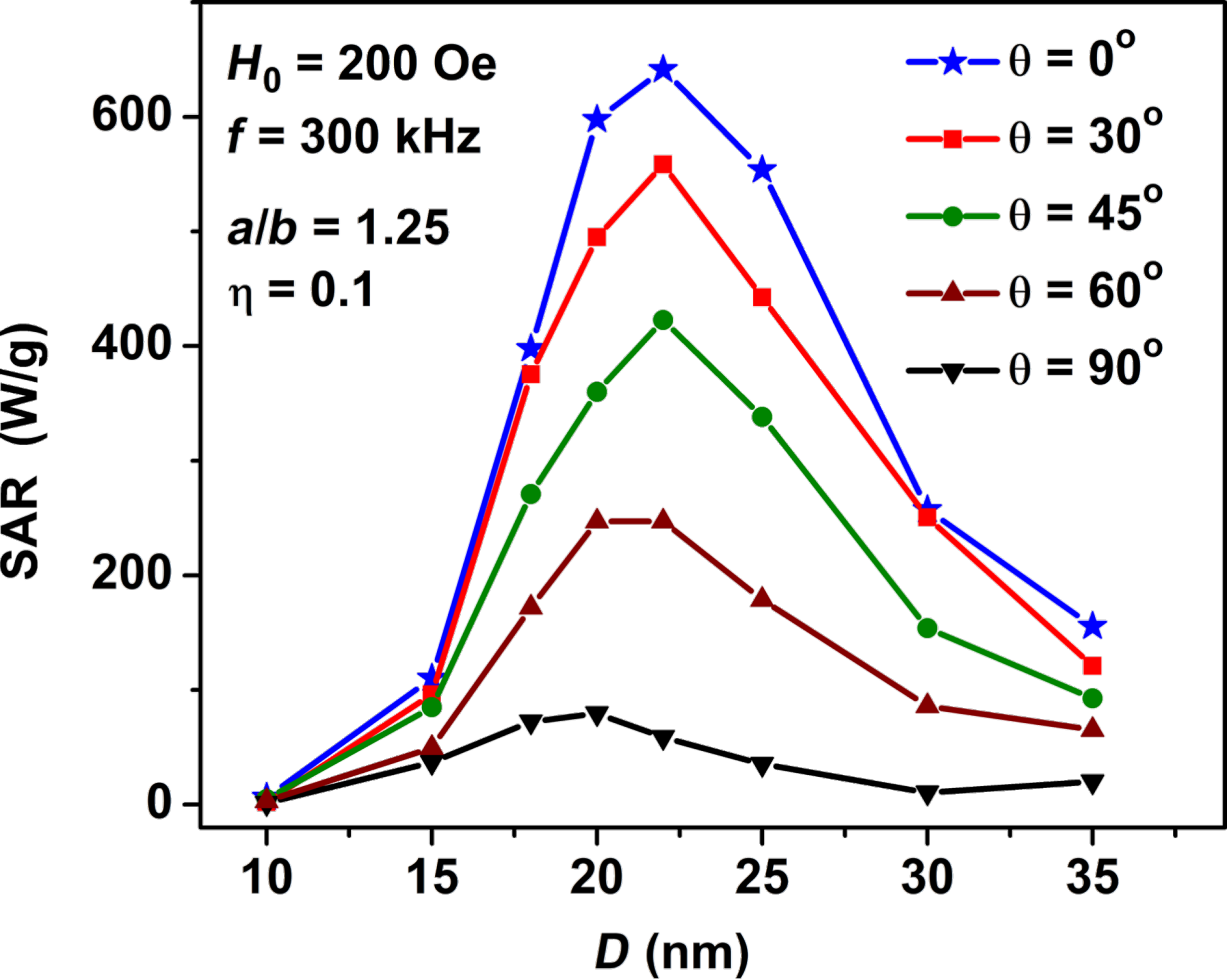
Dependence of the SAR of a dilute oriented assembly of clusters of spheroidal particles with aspect ratio *a*/*b* = 1.25 on the transverse particle diameter *D* for different directions of the ac magnetic field relative to the cluster orientation axis. The volume fraction of the particles in the cluster is η = 0.1.

For completeness, we also calculated the dependence of the SAR of a dilute assembly of oriented clusters on the direction of the ac magnetic field with respect to the cluster orientation axis. As Figure 5 shows, the SAR of the oriented assembly at its maximum rapidly decreases as a function of the inclination angle θ. In addition, the interval of optimal particle diameters gradually shifts to smaller sizes as a function of the angle θ.

## Conclusion

It is well known that in real assemblies of magnetic nanoparticles there is a certain distribution in the sizes and aspect ratios of particles [1–4]. In theoretical studies [18–23] spherical magnetic nanoparticles are usually considered. A theoretical study of assemblies of spherical nanoparticles with uniaxial [18–21,23,30] and cubic [25] magnetic anisotropy showed the existence of an interval of optimal nanoparticle diameters where the SAR of the assembly reaches a maximum at a given frequency and amplitude of an ac magnetic field. In this work, using numerical simulation, similar results were obtained for spheroidal magnetite nanoparticles in the range of aspect ratios *a*/*b* = 1.0–3.0. It is shown that due to the dominance of the shape anisotropy energy in the total magnetic anisotropy energy of the particles, an increase in the aspect ratio leads to a decrease in the SAR of randomly oriented assemblies of non-interacting nanoparticles. This is also accompanied by a shift of the interval of optimal particle diameters towards smaller sizes. Consequently, the use of elongated magnetite nanoparticles in magnetic hyperthermia, generally speaking, is disadvantageous. However, elongated magnetic nanoparticles can be relatively easily oriented in a sufficiently strong external magnetic field. In this work, it is shown that the maximum SAR value of an oriented assembly of non-interacting elongated magnetite nanoparticles increases approximately twice as compared to the maximum SAR value for an assembly of non-oriented nanoparticles at the same frequency and ac magnetic field amplitude. Thus, a dilute oriented assembly of elongated nanoparticles can compete fairly well with spherical nanoparticles. Nevertheless, it should be kept in mind that the strong MD interaction significantly reduces the SAR in dense clusters of nanoparticles. In this work it is shown that the SAR of a dilute oriented assembly of clusters decreases by almost an order of magnitude with an increase in the volume fraction of nanoparticles in a cluster in the range η = 0.04–0.2. One of the ways to prevent the aggregation of nanoparticles into dense clusters is to cover magnetic nanoparticles with nonmagnetic shells [23,25] of a thickness comparable to the transverse particle diameters.
